# Blunt-Nosed Viper (*Macrovipera lebetinus*) Venom: Proteomic Composition, Antioxidant Properties, and Anti-Alzheimer Assessment

**DOI:** 10.1021/acsomega.5c01428

**Published:** 2025-06-12

**Authors:** Noor Subhee Azz-Aldeen Azz-Aldeen, Hülya Tuba Kıyan, Sabriye Percin Ozkorucuklu, Mehmet Zülfü Yıldız, Naşit İğci, Mert Karış, Ebru Gurel Gurevin

**Affiliations:** † Department of Biology, Institute of Graduate Studies in Sciences, 37516 Istanbul University, Istanbul 34116, Türkiye; ‡ Department of Pharmacognosy, Faculty of Pharmacy, Anadolu University, Eskisehir 26470, Türkiye; § Department of Molecular Biology and Genetics, Faculty of Science, 37516 Istanbul University, Istanbul 34134, Türkiye; ∥ Zoology Section, Department of Biology, Faculty of Arts and Sciences, 563279 Adiyaman University, Adiyaman 02040, Türkiye; ⊥ Department of Molecular Biology and Genetics, Faculty of Arts and Sciences, Nevsehir Haci Bektas Veli University, Nevsehir 50300, Türkiye; # Program of Laboratory Technology, Acigöl Vocational School of Technical Sciences, Nevsehir Haci Bektas Veli University, Nevsehir 50300, Türkiye; ∇ Department of Biology, Faculty of Science, 37516 Istanbul University, Istanbul 34134, Türkiye

## Abstract

This study examines the antioxidant and enzyme (acetylcholinesterase, butyrylcholinesterase) inhibitory properties of crude and different solvent fractions of Macrovipera lebetinus obtusa venom and investigates the proteomic composition of its fractions (ethyl acetate, methanol, and water) employing liquid chromatography-tandem mass spectrometry (LC-MS/MS) analysis. The results revealed many peptide and protein families, suggesting considerable biological potential. Nineteen unique protein identifications belonging to eight families were achieved in the crude venom, along with additional identifications in particular fractions. Although major bioactive molecules in snake venoms are proteins and peptides, which dissolve readily in water-based solvents and can be separated by chromatography, here we also wondered if nonprotein components with different polarities and solubilization characteristics may also contribute to their activities. To this end, sequential fractionation with solvents of increasing polarity was performed. Utilizing techniques such as metal chelation, reducing power assessment, 2,2′-azinobis-3-ethylbenzothiazoline-6-sulfonic acid (ABTS), and free radical scavenging capabilities of 2,2-diphenyl-1-picrylhydrazyl (DPPH), it was determined that the methanol and ethyl acetate fractions demonstrated antioxidant activity, whereas other fractions displayed minimal effects. The methanol fraction of M. lebetinus obtusa venom exhibited significant metal chelation (60.77 ± 0.10%) and showed substantial antioxidant activity (reducing power) with an EC_50_ of 281.30 μg/mL. The ethyl acetate fraction also showed a reducing effect with an EC_50_ of 263.20 μg/mL. None of the fractions and crude venom showed antioxidant activity in the DPPH free radical scavenging assay and the ABTS^·+^ radical cation decolorization test. The ethyl acetate fraction exhibited significant acetylcholinesterase (AChE) inhibitory activity (75.39 ± 0.30%) comparable to donepezil (98.60 ± 0.10%); however, it displayed reduced butyrylcholinesterase (BuChE) inhibitory activity (20.65 ± 4.50%). The results highlight the potential of M. lebetinus obtusa venom fractions as sources of bioactive chemicals and the possible contribution of nonpolypeptide content to its activities.

## Graphical abstract

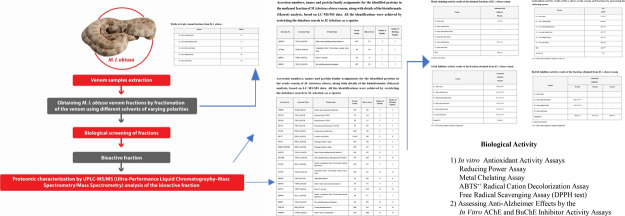

## 1 Introduction

Dementia, a neurodegenerative disease, is characterized by a progressive deterioration in cognitive functions. Neuropsychiatric symptoms include depression, agitation, and apathy. The patient increasingly depends on others for the performance of daily activities as the condition deteriorates. Alzheimer’s disease (AD) is the most common cause of dementia. Vascular dementia ranks as the second most common cause.^1^

The primary risk factor for dementia is age. Husband and Worsley^2^ report a prevalence of 20% in individuals aged 85 to 89 and 2% in those aged 65 to 69. In 2020, more than 50 million individuals were affected by dementia, with AD representing over 60% of all cases.^2–4^ AD impacts the central nervous system and is characterized as a chronic syndrome. Acetylcholinesterase (AChE) inhibitors function by decreasing the degradation of acetylcholine (ACh), thereby compensating for the depletion of central cholinergic neurons in AD and the resultant decrease in ACh levels. Brühlmann et al.^5^ indicate that AChE inhibitors are effectively employed in the treatment of AD.

Animal venom is a highly intricate and rich natural source of bioactive chemicals that interact with a diverse array of molecular targets and activities. Venoms are classified based on their source (snakes, scorpions, spiders, etc.) or their physiological effects (hemotoxins, neurotoxins, etc.). Animal venoms are generally aqueous solutions composed of a diverse array of elements, predominantly proteins and peptides. A single venom may comprise hundreds of unique compounds, referred to as toxins, whose primary objective is to kill or immobilize the target. Venoms may also contain various nontoxic molecules. Animal venoms significantly disrupt the circulatory, neurological, and muscular systems. Individuals afflicted by animal venoms experience acute illness and may ultimately succumb to death.^6,7^

One of the most notable animal secretion types that has captured the attention of scientists throughout history is snake venom.^6,8^ The venom of snakes in the Elapidae and Viperidae families presents a significant global health danger to humans; therefore, it is essential to investigate venom components to understand the pathophysiology of envenomation and determine the optimal response.^9^ Snake venom serves as a significant source of peptides and proteins exhibiting diverse pharmacological activities, positioning it as a promising candidate for drug development research. Research into snake venom proteins commenced in the 19th century, marking the beginning of modern biochemical studies of venoms. Nonetheless, a more thorough examination of snake venom proteins commenced in the late 1950s and early 1960s of the last century.^8^ The study of animal venoms has increased markedly, driven by a growing interest in the development of peptide-based therapeutics. Medications derived from snake venoms and proprietary diagnostic kits that have received approval from the Food and Drug Administration (FDA) are currently available in the market.^10,11^

Approximately 900 of the nearly 4177 snake species globally possess venom.^12^ Macrovipera lebetinus (Linnaeus, 1758), commonly known as the blunt-nosed viper, is the largest venomous viper species in Türkiye. Purification of many different proteins and peptides with distinct biological activities from M. lebetinus venom has proven its pharmacological potential.^13^ The main goals of peptide drug discovery studies include identifying novel peptides and proteins that influence various pathways, as well as discovering new variants of existing molecules with enhanced affinity and selectivity. While earlier research on snake venom in Türkiye aimed to conduct taxonomical comparisons,^14,15^ recent research has focused more on the biological and proteomic characterization of various viper venoms.^16–21^ Macrovipera lebetinus obtusa (Dwigubsky, 1832), belonging to the Viperidae family (Viperinae subfamily), is a subspecies of M. lebetinus and its distribution range covers Türkiye, Iraq, Afghanistan, Syria, Transcaucasia, Lebanon, Iran, Pakistan, Jordan. The venom of Viperids induces localized tissue necrosis, hypofibrinogenemia, and coagulation disorders in its prey.^16,18^

Snake venom typically comprises various proteins and peptides that have evolved to selectively interact with receptors, ion channels, or enzymes. The specific composition varies based on factors such as species, age, sex, and environmental factors.^22^ Some molecules in snake venoms can have detrimental effects, including neurotoxins and cardiotoxins. Relevant enzymes include phospholipase A_2_ (PLA_2_), phosphodiesterase (PDE), serine protease (SP), metalloprotease (MP), ?-amino acid oxidase (LAAO), acetylcholinesterase (AChE), 5′nucleotidase (5NT), adenosine diphosphatase (ADPase), adenosine triphosphatase (ATPase), and hyaluronidase. Inhibitors of enzymes, such as serine protease and metalloprotease inhibitors, are also found in snake venoms. Vasoactive peptides such as bradykinin-potentiating peptide (BPP) and C-type natriuretic peptide (CNP), disintegrin (DIS), vascular endothelial growth factor (VEGF), nerve growth factor (NGF), cysteine-rich secretory protein (CRISP), and C-type lectin (CLP) exemplify peptides and proteins that do not exhibit enzymatic activity.^22–24^

The BPP identified in the venom of Bothrops jararaca (jararaca) in 1965 represents a pivotal discovery illustrating the pharmacological potential of snake venoms and accelerating research in this domain. This ACE inhibitor peptide was utilized to develop captopril (Capoten), which is in use today following its approval by the FDA and the World Health Organization (WHO) for the management of heart disease and hypertension.^22^ Snake venom proteins are also valuable tools in scientific research. α-Bungarotoxin, a nonenzymatic protein in snake venom, is an α-neurotoxin present in the venom of Bungarus multicinctus. This protein facilitated the isolation of nicotinic acetylcholine receptors (nAChRs) through its binding to specific nAChRs. The clarification of the structural characteristics of this receptor has substantially advanced the literature on the creation of therapeutic medicines for autoimmune disorders, including myasthenia gravis and multiple sclerosis.^22^

The bioguided fractionation (BGF) technique using different solvents has been widely employed to isolate the bioactive components from plant extracts for several years. Based on this approach, potential drug prototypes were identified and the active components of medicinal plants which are used for different purposes, including antisnake venom, were determined.^25^ This efficient technique has recently been employed to extract fractions of active compounds from animal fluids such as amphibian skin secretion. The antiproliferative effects of skin secretions from Bufo bufo (common toad) and Rhinella marina (South American cane toad) on melanoma cell lines were investigated by using the aforementioned method, and their potential as prospective therapeutic agents for metastatic melanoma treatment was assessed.^26^

Bioactive molecules in snake venoms are mainly proteins and peptides, so that research on snake venoms generally focuses on purifying these proteins using liquid chromatography (ie, gel filtration, ion exchange, reversed-phase). However, venoms also contain nonpolypeptide molecules such as carbohydrates, biogenic amines, nucleosides, and lipids, which can be termed as minor components.^24,27^ Some of these compounds may have different polarities, resulting in their enrichment in various solvents other than water.

Although the venom proteome of M. lebetinus has been well-documented, there is limited information about the potential bioactivities of its metabolites. Moreover, there is no study investigating the AChE and butyrylcholinesterase (BuChE) inhibitory activity of M. lebetinus venom. This study aimed to examine the biological activities of M. l. obtusa crude venom and its fractions obtained by the BGF technique in terms of antioxidant and anti-Alzheimer (AChE/BuChE inhibition) potential, along with the proteomic characterization. The purpose of utilizing the BGF technique was to assess the activities of both proteins and other components, which may be enriched in solvents with different polarities.

## 2 Experimental Section

### 2.1 Venom Samples

A lyophilized pooled crude venom sample extracted from 10 adult M. l. obtusa individuals was used in all *in vitro* investigations. Snakes were collected from the southeastern region of Türkiye. Extraction and freeze-drying were performed as described before.^16^

### 2.2 Obtaining M. l. obtusa Venom Fractions

The chosen solvent is introduced in a sequence that advances from the least polar, beginning with *n*-hexane, to the most polar, concluding with water, throughout the fractionation process.^28,29^ 100 mg of crude venom from M. l. obtusa was sequentially fractionated by using maceration via the differential solubility method in six solvents of varying polarity: *n*-hexane, dichloromethane, ethyl acetate, methanol, and water. A rotary evaporator and a lyophilizer were employed to concentrate each recovered fraction. The temperature was maintained below −25 °C for 6–8 h (this step is necessary only if the product is freshly collected or has not been previously frozen), a primary drying pressure of 1.65–0.63 mbar was utilized, and a concluding drying pressure was reduced to 0.63–0.12 mbar. The yield of each solid fraction was preserved in a refrigerator at 4 °C until the bioassays were conducted.

### 2.3 Proteomic Characterization by UPLC-MS/MS (Ultra-Performance Liquid Chromatography–Mass Spectrometry/Mass Spectrometry) Analysis

The venom secretion of M. l. obtusa and its fractions (ethyl acetate, methanol, and water) were analyzed using a quadrupole-time-of-flight (Q-TOF) LC-MS/MS system, and the proteins were identified using a “bottom-up shotgun” proteomics approach. Tryptic peptides were initially separated using reversed-phase chromatography on a UPLC system directly connected to a mass spectrometer.^30^

#### 2.3.1 Enzymatic Digestion

In-solution digestion with trypsin was performed directly using crude venom or fraction samples, using a filter-aided sample preparation protocol (Expedon FASP protein digestion kit) based on a method described by Wiśniewski et al.^31^ Trypsin:sample (w/w) ratio was adjusted to 1:50 after the determination of the protein amount by Bradford’s method. Following peptide extraction, 1 μg of total peptide was injected into the column.

#### 2.3.2 LC-MS/MS Parameters

The analysis was conducted using a SYNAPT Xevo G2-XS system (Waters) in conjunction with an ACQUITY UPLC M-Class. The column temperature was set to 45 °C and equilibrated with 97% mobile phase A (0.1% formic acid (FA) in LC-MS grade water from Merck) prior to the analyses. Peptides were initially isolated from the trap column (Symmetry C_18_ 5 μm, 180 μm i.d. × 20 mm) using a linear gradient elution (4 to 40% acetonitrile 0.1% (v/v) FA) onto the analytical column (Charged Surface Hybrid (CSH) C_18_, 1.7 μm, 75 μm i.d. × 250 mm) at a flow rate of 0.2 μL/min. Additionally, 100 fmol/μL Glu-1-fibrinopeptide B (Glu-fib) served as a lock mass reference, introduced to the source at a flow rate of 0.5 μL/min with a 60-s interval. The mass spectrometry instrument operated in positive ion mode. A novel method of data acquisition, termed SONAR, was employed for the collection of MS data. During SONAR mode, the quadrupole was continuously scanned across the *m*/*z* range of 400 to 900, with a quadrupole transmission width of 24 Da maintained. Sequential MS and MS/MS scans were performed with a low collision energy (CE) of 14 V and a high CE of 40 V. The cycle time measured 0.5 s. All ions within the *m*/*z* range of 50 to 1950 were fragmented collectively, without preselection of precursor ions, while operating in sensitivity mode. The data for fragmented ions were synchronized with their respective precursors according to retention time.

#### 2.3.3 Data Management

The Progenesis-QI for proteomics software (v4.1, Waters) was employed for the quantitative evaluation of peptide features and for protein identification. The Glu-fib peptide with *m*/*z* 785.8426 served as the calibrant, and the sample sets were modified according to the total ion intensity. The processing parameters were established with high and low energy thresholds of 60 and 10, respectively. Mass data were imported into Progenesis QI, and analysis was performed based on the following criteria: a minimum of two fragmented ion matches per peptide, at least five fragment ion matches per protein, a minimum of one unique peptide per protein, allowance for one missed cleavage in tryptic digestion, fixed modification as carbamidomethyl C, variable modifications including oxidation M and deamidation for N and Q, and a false discovery rate (FDR) of ≤1%. Only ions with charges of +2 and +3 were selected. Normalization among samples was performed utilizing total ion intensity. Expression levels and *p* and *q* values were calculated using the statistical package within Progenesis QI for proteomics. Protein normalization was performed based on relative quantitation utilizing nonconflicting peptides.

### 2.4 Antioxidant Activity Assays

Four different methods were used to assess the antioxidant capacity of M. l. obtusa crude venom and fractions. All the experiments were performed at least three times.

#### 2.4.1 Reducing Power Activity

The enhanced technique for assessing the reducing power of samples was employed.^32,33^ Samples were mixed with 500 μL of phosphate buffer (200 mM, pH 6.6) and 500 μL of 1% potassium ferricyanide at different concentrations. The mixtures were incubated at 50 °C for 20 min. After incubation, 500 μL of 10% trichloroacetic acid was added to the mixtures, which were subsequently centrifuged at 4000 rpm for 10 min. The absorbance of the resulting solution was measured at 700 nm following the combination of 500 μL of the upper layer with 200 μL of 0.1% ferric chloride and 500 μL of distilled water. Increased reaction absorbance led to greater reducing power. EC_50_ denotes the concentration that yields an absorbance of 0.5 at a wavelength of 700 nm. A lower EC_50_ indicates a greater reducing power.

#### 2.4.2 *In Vitro* Metal Chelating Assay

The capacity of the samples to chelate ferrous ions was assessed using a modified version of the method.^32^ 40 μL of a 2 mM FeCl_2_ solution were combined with 1480 μL of methanol and 400 μL of the samples. 80 μL of 5 mM ferrozine was introduced to the mixture to initiate the reaction, which was thereafter allowed to run for 15 min at ambient temperature. The absorbance of the reaction mixture was measured at 562 nm. EDTA served as the positive control for the assay. The subsequent formula was employed to ascertain the ratio of inhibition of ferrozine-Fe^2+^ complex formation:

$$\mathrm{Metal}\;\mathrm{Chelating}\;\mathrm{capacity}\;(\%)=\frac{A_{\mathrm{Control}}-A_{\mathrm{Sample}}}{A_{\mathrm{Control}}}\times 100$$

#### 2.4.3 ABTS^·+^ Radical Cation Decolorization Assay

The oxidation of 2,2′-azinobis-3-ethylbenzothiazoline-6-sulfonic acid (ABTS) with potassium persulfate results in the formation of the ABTS^·+^ radical cation. The reagent, generated fresh prior to each investigation, was diluted with ethanol to achieve an absorbance of 0.70 (±0.02) at a wavelength of 734 nm for the assessment of antioxidant activity. To facilitate the pertinent reaction, 990 μL of reagent solution was introduced to the A-Blank and A-sample, which contained 10 μL of sample, along with the sample solution at an equivalent concentration. The mixture was allowed to stand for 5 min, after which its absorbance values at 734 nm were recorded. In this study, ascorbic acid (vitamin C) served as a positive control, whereas ethanol functioned as a blank for comparative analysis. Following spectrophotometric analysis, the percentage elimination of the ABTS^·+^ radical cation was computed using the equation provided below. In the equation, *A*_control_ denotes the absorbance value of the ABTS reagent solution, whereas *A*_sample_ denotes the absorbance of the reagent solution that includes the sample formulation.^32,33^

$$\mathrm{Total}\;\mathrm{Antioxidant}\;\mathrm{Activity}\;(\%)=\frac{A_{\mathrm{Control}}-A_{\mathrm{Sample}}}{A_{\mathrm{Control}}}\times 100$$

#### 2.4.4 Free Radical Scavenging Assay (DPPH Test)

The scavenging effects of the samples on 2,2-diphenyl-1-picrylhydrazyl (DPPH) free radicals were assessed using a modified methodology.^32,33^ The free radical scavenging activities of the samples were quantified as a percentage of inhibition, computed using the equation shown below. *A*_control_ represents the absorbance of the control (which includes all reagents except the test substance), while *A*_sample_ denotes the absorbance of the sample with added DPPH. The IC_50_ values were derived by graphing the DPPH scavenging percentage of the sample in relation to its concentration.

$$\mathrm{Inhibition}\;(\%)=\frac{A_{\mathrm{Control}}-A_{\mathrm{Sample}}}{A_{\mathrm{Control}}}\times 100$$

### 2.5 Assessing Anti-Alzheimer Effects by the *In Vitro* AChE and BuChE Inhibitor Activity Assays

The Ellman test was employed to assess the inhibitory activity of the test compounds on butyrylcholinesterase (BuChE) and acetylcholinesterase (AChE) enzymes.^34^ Modifications to the procedure were implemented to enhance colorimetric clarity, and the revised method was utilized. This study utilized BuChE (extracted from horse serum, E.C.3.1.1.8.), AChE (derived from Electrophorus electricus, E.C.3.1.1.7.), Ellman’s reagent, buffer solutions (potassium dihydrogen phosphate, potassium hydroxide), 5,5′-dithiobis (2-nitrobenzoic acid) (DTNB), gelatin, acetylcholine iodide (ATC), sodium hydrogen carbonate, butyrylcholine iodide (BTC), dimethyl sulfoxide (DMSO), and donepezil. Experiments to assess the inhibitory actions of the test compounds on AChE and BuChE enzymes were conducted in 96-well plates. Subsequently, solutions of 2.5 μg/mL AChE and BuChE enzymes, 0.075 M ATC and BTC, 0.01 M DTNB, and 0.1 M phosphate buffer at pH 8, together with test solutions at varying doses, were produced for experimental usage.

The solutions were combined to create two distinct test solutions. The initial test solution was generated by combining 70 μL of phosphate buffer solution, 20 μL of DTNB solution, and 20 μL of enzyme solution (distinct test solutions were formulated for both AChE and BuChE) for each well. The alternative test solution was made by combining 70 μL of phosphate buffer solution with 10 μL of either ATC or BTC solution, with a distinct test solution created for each of ATC and BTC in every well. Test solutions were formulated in proportions adequate for all 96 wells. Initially, 20 μL of the test solution and 110 μL of the first test solution were dispensed onto the 96-well plate in quadruplicate for each concentration, stirred for 5 min, and incubated for 15 min at 25 °C. Subsequent to the incubation, 80 μL of the secondary test solution was introduced to each well, followed by a quick mixing procedure lasting 30 s. After the mixing procedure, the initial spectrophotometric measurement was conducted at a wavelength of 412 nm using a microplate reader (BioTek-Synergy H1Microplate Reader). Following a 5 min wait for the reaction to progress, the second spectrophotometric measurement was conducted at the conclusion of the interval. The % inhibition rates were computed by determining the difference in absorbance values between the two measurements, as indicated by the equation below. The elucidations of the abbreviations employed in the equation are as follows: A­(C): Difference in absorbance reading of the control well; C: Control (well without test solution); B: Blank (well without test solution and substrate); A­(B): Difference in absorbance reading of the blank well; A­(I): Difference in absorbance reading of the test solution.

$$\%\mathrm{Inhibition}=\frac{\left[(A(C)-A(B))-(A(I)-A(B))\right]}{\left(A(C)-A(B)\right)}\times 100$$

### 2.6 Statistical Analysis

All data obtained were evaluated statistically (one-way ANOVA test) using GraphPad Prism version 8.0. All measurements were done for at least three replicates. The results were presented as Mean ± SD. A value of *p* < 0.05 was considered statistically significant.

## 3 Results and Discussion

### 3.1 Yields of Fractions

The yields (w/w %) of the fractions from the crude venom of Macrovipera lebetinus obtusa were assessed according to the dry weight of the venom (Table 1). The yield was increased as the polarity of the solvent increased, due to the enrichment of proteins in methanol and water fractions. Because other nonproteinaceous minor constituents make up a smaller percentage of snake venoms,^27^ the yields are low in nonaqueous fractions.

**Table 1:** Yields of Crude Venom Fractions from M. l. obtusa

fractions	yields %
*M. l. obtusa n*-hexane fraction	0.90
*M. l. obtusa* dichloromethane fraction	1.30
*M. l. obtusa* ethyl acetate fraction	3.10
*M. l. obtusa* methanol fraction	8.80
*M. l. obtusa* water fraction	12.90

### 3.2 Analysis of the Selected Fractions Using Liquid Chromatography-Tandem Mass Spectrometry (LC-MS/MS)

The chemical (proteomic) profile analysis of M. l. obtusa crude venom and its selected fractions with higher yields (ethyl acetate, methanol, and water) performed via LC-MS/MS identified a substantial number of components (Tables 2–5). As snake venoms serve as important sources for the advancement of peptide-based pharmaceuticals, performing their biochemical and proteomic characterization is crucial for assessing their potential. Mass spectrometry is a critical technique for identifying and characterizing venom constituents. In this study, LC-MS/MS analysis elucidated the diverse proteins and peptides, identifying a total of 19 unique proteins belonging to eight different families (CLP, SP, NGF, LAAO, DIS, MP, PDE, and 5NT) in M. l. obtusa crude venom (Table 2). Furthermore, the ethyl acetate fraction of M. l. obtusa venom yielded a total of two peptide/protein families (MP and CLP), as indicated in Table 3, and the methanol fraction of M. l. obtusa venom exhibited an identical count of four peptides/proteins from two families (MP and SP) (Table 4). Additionally, the aqueous fraction of M. l. obtusa venom revealed six peptide/protein families (SP, DIS, NGF, LAAO, MP, and CLP) based on 16 different protein identifications, as indicated in Table 5.

**Table 2:** Accession Numbers, Names, and Protein Family Assignments for the Identified Proteins in the Crude Venom of M. lebetinus obtusa, Along with Details of the Bioinformatic (Mascot) Analysis, Based on LC-MS/MS Data^a^

accession no.	accession name	protein name	protein family	Mascot score	number of peptides	number of matching peptides
C0HKZ7	SLMB_MACLB	Snaclec macrovipecetin subunit beta	CLP	262	3	3
E0Y418	VSP1_MACLB	Serine protease VLSP-1	SP	418	8	7
E0Y420	VSP3_MACLB	Serine protease VLSP-3	SP	211	2	1
E0Y421	VSPY_MACLB	Chymotrypsin-like protease VLCTLP	SP	381	9	5
P25428	NGFV_MACLB	Venom nerve growth factor	NGF	500	8	7
P81375	OXLA_MACLB	L-amino-acid oxidase	LAAO	560	8	8
P83253	DID1A_MACLB	Disintegrin lebein-1-alpha	DIS	268	4	4
Q3BK13;P0C6B0	DID2A_MACLB	Disintegrin lebein-2-alpha	DIS	242	4	4
Q3ZD74	VM1L4_MACLB	Snake venom metalloproteinase lebetase-4	MP	343	8	7
Q4VM08	VM3VA_MACLB	Zinc metalloproteinase-disintegrin-like VLAIP-A	MP	1265	21	19
Q7T045	SLLC1_MACLB	Snaclec coagulation factor X-activating enzyme light chain 1	CLP	325	5	5
Q7T046	VM3CX_MACLB	Coagulation factor X-activating enzyme heavy chain	MP	191	24	22
Q8JH85	VSPA_MACLB	Alpha-fibrinogenase	SP	708	11	9
Q9PT40	VSPH2_MACLB	Snake venom serine protease homologue 2	SP	475	9	7
Q9PT41	VSPF5_MACLB	Factor V activator	SP	1390	28	26
Q696W1	SLLC2_MACLB	Snaclec coagulation factor X-activating enzyme light chain 2	SP	517	5	5
Q98995	VM2L2_MACLB	Zinc metalloproteinase/disintegrin	MP	900	16	15
W8E7D1	PDE_MACLB	Venom phosphodiesterase	PDE	104	16	16
W8EFS0	V5NTD_MACLB	Snake venom 5′-nucleotidase	5NT	1606	24	24

a All the identifications were achieved by restricting the database search to M. lebetinus as a species.

**Table 3:** Accession Numbers, Names, and Protein Family Assignments for the Identified Proteins in the Ethyl Acetate Fraction of M. lebetinus obtusa Venom, Along with Details of the Bioinformatic (Mascot) Analysis, Based on LC-MS/MS Data^a^

accession no.	accession name	protein name	protein family	Mascot score	number of peptides	number of matching peptides
Q3ZD74	VM1L4_MACLB	Snake venom metalloproteinase lebetase-4	MP	6	1	1
Q7T046	VM3CX_MACLB	Coagulation factor X-activating enzyme heavy chain	MP	6	1	1
Q98995	VM2L2_MACLB	Zinc metalloproteinase/disintegrin	MP	141	2	1
A0A0C5DM02	A0A0C5DM02_MACLB	C-type lectin-like protein 3B	CLP	7	1	1

a All the identifications were achieved by restricting the database search to M. lebetinus as a species.

**Table 4:** Accession Numbers, Names, and Protein Family Assignments for the Identified Proteins in the Methanol Fraction of M. lebetinus obtusa Venom, Along with Details of the Bioinformatic (Mascot) Analysis, Based on LC-MS/MS Data^a^

accession no.	accession name	protein name	protein family	Mascot score	number of peptides	number of matching peptides
Q3ZD74	VM1L4_MACLB	Snake venom metalloproteinase lebetase-4	MP	272	3	3
Q7T046	VM3CX_MACLB	Coagulation factor X-activating enzyme heavy chain	SP	195	2	2
Q9PT41	VSPF5_MACLB	Factor V activator	SP	6	1	1
Q98995	VM2L2_MACLB	Zinc metalloproteinase/disintegrin	MP	351	5	5

a All the identifications were achieved by restricting the database search to M. lebetinus as a species.

**Table 5:** Accession Numbers, Names, and Protein Family Assignments for the Identified Proteins in the Water Fraction of M. lebetinus obtusa Venom, Along with Details of the Bioinformatic (Mascot) Analysis, Based on LC-MS/MS Data^a^

accession no.	accession name	protein name	protein family	Mascot score	number of peptides	number of matching peptides
E0Y418	VSP1_MACLB	Serine protease VLSP-1	SP	351	8	7
E0Y419	VSPBF_MACLB	Beta-fibrinogenase	SP	298	5	5
P0C6B0;Q3BK13	DID5B_MACLO	Disintegrin VLO5B	DIS	6	1	1
P25428	NGFV_MACLB	Venom nerve growth factor	NGF	203	3	3
P81375	OXLA_MACLB	L-amino-acid oxidase	LAAO	386	4	4
Q3ZD74	VM1L4_MACLB	Snake venom metalloproteinase lebetase-4	MP	7	1	1
Q4VM08	VM3VA_MACLB	Zinc metalloproteinase-disintegrin-like VLAIP-A	MP	280	5	5
Q7T046	VM3CX_MACLB	Coagulation factor X-activating enzyme heavy chain	MP	958	15	15
Q8JH85;E0Y420	VSPA_MACLB	Alpha-fibrinogenase	SP	436	5	5
Q9PT40	VSPH2_MACLB	Snake venom serine protease homologue 2	SP	498	7	6
Q9PT41	VSPF5_MACLB	Factor V activator	SP	807	17	16
Q696W1	SLLC2_MACLB	Snaclec coagulation factor X-activating enzyme light chain 2	CLP	21	3	3
Q98995	VM2L2_MACLB	Zinc metalloproteinase/disintegrin	MP	656	10	10
W5XCJ6;B4XSY9;B4XSZ0	SLCIB_MACLB	Snaclec lebecin subunit beta	CLP	18	3	3
E0Y418	VSP1_MACLB	Serine protease VLSP-1	SP	351	8	7

a All the identifications were achieved by restricting the database search to M. lebetinus as a species.

Previous proteomic characterizations provided insight into the venom arsenal of M. l. obtusa from Türkiye. Two of these studies employed a venomics approach by using HPLC fractionation followed by sodium dodecyl sulfate-polyacrylamide gel electrophoresis (SDS-PAGE) separation, and subsequent protein identification based on LC-MS/MS-derived peptide sequences.^35,36^ The following protein families were reported in the aforementioned studies: MP, SP, PLA_2_, LAAO, CLP, CRISP, DIS, BPP/CNP, Kunitz-type SP inhibitor, and tripeptide MP inhibitor. Moreover, MP, SP, PLA_2_, LAAO, CLP, CRISP, DIS, VEGF, NGF, hyaluronidase, and SP inhibitor were identified in another study utilizing a different methodology using a combination of two-dimensional polyacrylamide gel electrophoresis (2D-PAGE) and matrix assisted laser desorption ionization-time-of-flight (MALDI-TOF) mass spectrometry-based peptide mass fingerprinting.^16^ Anatolian M. l. obtusa venom was also shown to have significant MP/SP, PLA_2_, LAAO, PDE, and hyaluronidase activities.^37^ Macrovipera lebetinus obtusa venoms from other geographical regions were also investigated. Pla et al.^38^ utilized a venomics approach and identified MP, SP, PLA_2_, LAAO, CLP, CRISP, DIS, BPP, and DC-fragment in M. l. obtusa venom from Russia. Using a similar method, Sanz et al.^39^ reported SP, CLP, MP, LAAO, BPP, DIS, CRISP, PLA_2_, NGF, and DC-fragment in M. l. obtusa venom from Armenia.

Based on the current knowledge, all the protein families identified in the present study were also reported from M. lebetinus venom previously.^13,16,36,39^ Although the data of the study does not add new information on the proteomic composition of M. l. obtusa venom, we aimed to reanalyze and characterize our pooled venom sample in case of any variation, as well as to assess the solubilities of individual proteins if different polarity solvents have significant protein content. As expected, water fraction (as the most polar protic solvent) was the main protein extract. Limited number of proteins found in ethyl acetate (a polar aprotic solvent) and methanol (a polar protic solvent) fractions may be due to their slight solubility in these solvents or occasional contamination during the sequential liquid–liquid extraction process. Polar protic solvents are superior in dissolving proteins. However, as the polarity of the solvent decreases, the solubility of the protein also generally decreases.^40^ Different molecule groups in venom that may be found in solvents other than water, and their potential contribution to biological activities, are discussed later.

### 3.3 Antioxidant Activity of Test Substances

#### 3.3.1 Results of Metal Chelating Activity

The metal ion chelating activity of M. l. obtusa venom fractions was assessed based on their ability to compete with ferrosine for binding Fe^2+^ ions in solution, with EDTA serving as a positive control. Results indicated that EDTA at a concentration of 500 μg/mL exhibited an antioxidant activity of 99.08%. In comparison, the M. l. obtusa methanol fraction demonstrated significant antioxidant activity with inhibition rates of 60.77% (Table 6). Crude venom showed negligible activity, whereas other fractions showed no detectable activity.

**Table 6:** Metal Chelating Activity Results of M. l. obtusa Crude Venom and the Fractions^a^

fractions	antioxidant activity inhibition % 500 μg/mL
*M. l. obtusa* venom	5.34 ± 0.20
*M. l. obtusa n*-hexane fraction	-
*M. l. obtusa* dichloromethane fraction	-
*M. l. obtusa* ethyl acetate fraction	-
*M. l. obtusa* methanol fraction	60.77 ± 0.10
*M. l. obtusa* water fraction	-
EDTA	99.08 ± 0.20

a (-): Activity was not observed.

#### 3.3.2 Results of Reducing Power Measurement

The reducing power measurement of the extracts was conducted using the potassium ferrocyanide reducing method. The reducing power of the samples, attributed to their capacity to donate hydrogen, is typically linked to the presence of reductones.^41^ EC_50_ values derived from reducing power measurements were compared to those of butylated hydroxytoluene (BHT) (EC_50_ = 186.80 μg/mL) and ascorbic acid (EC_50_ = 92.80 μg/mL) as potent antioxidant molecules. The M. l. obtusa ethyl acetate and methanol fractions exhibited the highest reducing power activities with an EC_50_ of 263.20 and 281.30 μg/mL, respectively, while the dichloromethane extract demonstrated the lowest activity with an EC_50_ value of 490.20 μg/mL (*p* < 0.0001) (Table 7).

**Table 7:** Antioxidant Activity Results of M. l. obtusa Crude Venom and the Fractions by Measuring the Reducing Power^a^

fractions	EC_50_ (μg/mL)
*M. l. obtusa* venom	373.30****
*M. l. obtusa n*-hexane fraction	446.40****
*M. l. obtusa* dichloromethane fraction	490.20****
*M. l. obtusa* ethyl acetate fraction	263.20****
*M. l. obtusa* methanol fraction	281.30****
*M. l. obtusa* water fraction	403.40****
BHT	186.80****
Ascorbic acid	92.80

a ****; *p* < 0.0001 statistically significant difference compared to Ascorbic acid.

#### 3.3.3 Results of ABTS^·+^ Radical Cation Decolorization Activity

This method involved the relative measurement of antioxidative substances retained by the ABTS^·+^ radical cation in comparison to standard amounts of trolox, a synthetic antioxidant and water-soluble vitamin E analogue. Measurements were conducted by spectrophotometrically assessing the color reduction of the ABTS^·+^ radical, a stable blue/green compound. The interaction of ABTS with potassium persulfate resulted in the formation of the blue-green ABTS^·+^ chromophore. The crude venom of M. l. obtusa, and its *n*-hexane, dichloromethane, ethyl acetate, methanol, and water fractions exhibited no antioxidant activity as determined by the ABTS^·+^ radical cation decolorization method.

#### 3.3.4 Results of the DPPH Free Radical Scavenger Activity

The DPPH assay is used to evaluate the antioxidant activities of molecules by measuring the scavenging of DPPH radical, which represents the mechanism of antioxidant chemical compounds mitigating lipid peroxidation. It is among the most concise and economical approaches to assess hydrogen donor potential. Numerous radical ions in nature are neutralized by various chemicals via distinct chemical processes. DPPH, a persistent organic radical, is utilized to assess the antioxidant activity of various extracts and compounds.^42^ Macrovipera lebetinus obtusa crude venom, *n*-hexane, dichloromethane, ethyl acetate, methanol, and water fractions had no detectable antioxidant activity as determined by the DPPH free radical scavenging technique.

### 3.4 *In Vitro* Enzymatic Activity Assays

#### 3.4.1 AChE Inhibitory Activity Results

The method developed by Ellman et al.^34^ utilizes colorimetric measurement to quantify the release of thiocholine into the environment during the hydrolysis of ACh by AChE. The method involves comparing the absorbance of a fixed dose of AChE with a specific volume of substrate of known concentration to the absorbance observed upon the addition of the inhibitory compound or extract.^43^ The results of AChE enzyme inhibitor activity indicated that, when compared to the reference substance donepezil, the ethyl acetate fraction of M. l. obtusa crude venom exhibited a high inhibitory activity of 75.39% at a concentration of 500 μg/mL (*p* < 0.0001). In contrast, other fractions of M. l. obtusa crude venom demonstrated lower AChE inhibitory activities (Table 8).

**Table 8:** AChE Inhibitor Activity Results of M. l. obtusa Crude Venom and the Fractions^a^

fractions	concentration inhibition% 500 μg/mL
*M. l. obtusa* venom	33.96 ± 0.40****
*M. l. obtusa n*-hexane fraction	62.22 ± 0.10****
*M. l. obtusa* dichloromethane fraction	64.17 ± 0.10****
*M. l. obtusa* ethyl acetate fraction	75.39 ± 0.30****
*M. l. obtusa* methanol fraction	54.65 ± 0.20****
*M. l. obtusa* water fraction	49.76 ± 0.10****
Donepezil HCl	98.60 ± 0.10

a ****; *p* < 0.0001 statistically significant difference compared to Donepezil HCl.

#### 3.4.2 BuChE Inhibitory Activity Results

BuChE enzyme inhibitor activity results indicate that, when compared to the reference substance donepezil, the ethyl acetate fraction of M. l. obtusa crude venom exhibited lower inhibitory activity at a concentration of 400 μg/mL (20.65%), while the methanol fraction demonstrated much lower activity (15.45%) (*p* < 0.0001). Other fractions of M. l. obtusa crude venom showed no BuChE inhibitory activity at concentrations of 100, 200, and 400 μg/mL (Table 9).

**Table 9:** BuChE Inhibitor Activity Results of M. l. obtusa Crude Venom and the Fractions^a^,^b^

	concentration inhibition%		
fractions	400 μg/mL	200 μg/mL	100 μg/mL
*M. l. obtusa* venom	-	-	-
*M. l. obtusa* ethyl acetate fraction	20.65 ± 4.50****	-	-
*M. l. obtusa* methanol fraction	15.45 ± 4.80****	-	-
*M. l. obtusa* water fraction	-	-	-
Donepezil HCl	90.20 ± 1.20	83.12 ± 4.60	76.04 ± 4.10

a (-): Activity was not observed.

b ****; *p* < 0.0001 statistically significant difference compared to Donepezil HCl.

### 3.5 Evaluation of Antioxidant and AChE/BuChE Inhibitory Activities of M. l. obtusa Venom Fractions

This study examines the antioxidant properties and the AChE and BuChE inhibitory effects of various fractions of M. l. obtusa crude venom extract, including *n*-hexane, dichloromethane, ethyl acetate, methanol, and water. It also addresses the potential pharmacological applications focusing on Alzheimer’s disease (AD), emphasizing the research focus on M. l. obtusa crude venom and the proteins/peptides, as well as other minor constituents derived from it.

AChE is the primary enzyme implicated in the hydrolysis of ACh and contributes to the development of AD. The cholinergic hypothesis posits that the restoration of ACh levels, which diminish as AD advances, can delay the deterioration of cognitive function. Recent research indicates that increasing acetylcholine levels in the synaptic region through AChE inhibitors can enhance cognitive function and reduce symptoms in neuropsychiatric patients with AD.^44^

Cholinesterase represents a critical target for the treatment of AD. The degeneration of cholinergic neurons in the brain is the principal factor contributing to the increase in cognitive impairments observed in AD patients. Amyloid beta (Aβ) plaques and neurofibrillary tangles represent the primary neuropathological features observed in patients with AD. AChE accelerates the aggregation of amyloid-beta (Aβ) in cholinergic terminals.^5,45,46^ AChE inhibitors have been utilized to reduce the rate of ACh degradation and potentially enhance neuronal function. Along with its different biological roles, BuChE cooperates with AChE to regulate ACh metabolism. The differential distribution of AChE and BuChE in the brain suggests potential biological interactions between these two enzymes that may be significant. BuChE’s potential to substitute AChE in regulating cholinergic transmission suggests its viability as an alternative therapeutic target in AD.^47,48^

The pathophysiology of AD is intricately linked to neuronal damage caused by free radicals, which is exacerbated by oxidative stress and the accumulation of metals in the brain. Patients with AD exhibit varying concentrations of iron, ferritin, and transferrin in the cerebral cortex and hippocampus, alongside disruptions in iron homeostasis. Iron contributes to the accumulation of Aβ. Chelation of iron and other redox-active metals, such as copper, iron represents a valuable therapeutic strategy for AD when used with a cholinergic inhibitor.^49,50^

In line with this knowledge, we also assessed the antioxidant potential of M. l. obtusa venom fractions. Among all the extracts, the methanol and ethyl acetate fractions stand out in terms of antioxidant (iron chelating and reducing) activity. The methanol fraction exhibited notable antioxidant (iron chelating) activity, and the results revealed that the ethyl acetate (EC_50_ = 263.20 μg/mL and methanol (EC_50_ = 281.30 μg/mL fractions (500 μg/mL) exhibited the highest iron (ferric) reducing capacities, comparable to BHT (186.80 μg/mL) and ascorbic acid (92.80 μg/mL). The results of DPPH and ABTS^·+^ assays indicated that M. l. obtusa crude venom and its fractions had no free radical scavenging activity.

Peptides derived from animal sources, especially snake venom, may demonstrate antioxidant and neuroprotective properties. Recent studies highlight the importance of exploring animal secretions as a potential source of antioxidant peptides.^51^ Dematei et al.^52^ showed that the peptide BmT-2, derived from the venom of Bothrops moojeni, is the first identified tryptophyllin in snakes, exhibiting antioxidant activity *in vitro* and in cellular experiments. Another study demonstrated that the BPP, found in the venom of Bothrops jararaca, exhibited a protective effect against hydrogen peroxide (H_2_O_2_)-induced toxicity in the human neuroblastoma (SH-SY5Y) cell line, at a concentration of 0.1 μg/mL.^53^ Preventing mitochondrial alterations and maintaining plasma membrane fluidity may enhance neuroprotection. Additionally, the DPPH assay indicated that this peptide did not act as a scavenger of reactive species. Thus, BPP’s neuroprotective effect is unrelated to its ability to absorb unpaired electrons and thereby reduce the abundance of reactive oxygen species in the environment. By modifying the composition of antioxidant compounds such as polyamines, BPP can enhance neuroprotection while inhibiting changes in lipid peroxidation and mitochondrial membrane permeability.^54,55^

Apart from snake venoms, additional research indicated that the peptide antioxidin-I, found in the skin secretion of Physalaemus nattereri (a species of frog), exhibits neuroprotective characteristics in human microglia by diminishing the production of reactive oxygen species induced by hypoxia.^56^ Salamandrin-l, a peptide found in the epidermal secretions of Salamandra salamandra, exhibits *in vitro* radical scavenging action in ABTS and DPPH assays.^57^

Although we did not detect BPPs (and other small peptides) in any of the fractions or crude venom through our proteomic analysis workflow, it has been shown that M. l. obtusa venom contains these peptides in considerable amounts.^36,38,39^ As these are small peptides with mostly uncharged residues, it is possible that they dissolve in different solvents to a certain extent or can move more easily between solvents during sequential extraction.

The gradual degeneration of neurons is a characteristic feature of neurodegenerative disorders that impact numerous individuals. Oxidative stress, particularly reactive oxygen species (ROS), significantly contributes to the progression of various disorders, as demonstrated by the identification of lipid, protein, and DNA oxidation products in *in vivo* studies.^58,59^ ROS are autonomous molecules that comprise at least one oxygen atom and possess one or more unpaired electrons. They encompass reactive nitrogen species (RNS), including nitric oxide (NO), and oxygen free radicals such as superoxide anion radical (O_2_^·–^), hydroxyl radical (OH^·^), hydroperoxyl radical (HOO^·^), and singlet oxygen (O^·2^).^58,60^ The intricate processes of neutralizing free radicals, charge transfer mechanisms, and chemical reactions are generally implicated in the oxidative cascade reactions that result in cellular damage, which antioxidant molecules can mitigate or inhibit.^61^ Antioxidants are substances created by the body, such as glutathione, uric acid, and ubiquinone, as well as those obtained from the diet, including vitamins C, E, and A, and flavonoids. These antioxidant molecules mitigate the detrimental effects of free radicals.^62^

The results of the present study showed that the ethyl acetate fraction of M. l. obtusa venom showed significant AChE activity and slight BuChE activity. It also had the highest iron-reducing but not chelating activity. Although antioxidant activities are not directly related to the AChE inhibitory action, they may have secondary benefits in preventing AD, as discussed before. However, more detailed analyses should be carried out to find out if it is the same molecule with both iron-reducing and AChE/BuChE inhibitory activities. The methanol fraction showed both iron-reducing and chelating activities, but its enzyme activities were lower than those of the ethyl acetate fraction.

## 4 Conclusions

It is mostly composed of proteins and peptides that make up snake venom. On the other hand, venoms include a variety of substances that are considered to be minor ingredients. These substances include lipids, carbohydrates, amines, nucleosides, free bases, different carboxylic acids, amino acid residues, and nucleic acids.^27,63^ When compared to other fractions and crude venom, the ethyl acetate fraction of M. l. obtusa crude venom exhibited increased levels of AChE and decreased levels of BuChE inhibitory activity. This was the conclusion that we reached based on our findings. As a result of the observations, it has been determined that AChE inhibitors, which are employed to decrease the pace at which ACh is degraded, are particularly present in the ethyl acetate fraction. Additionally, the methanol fraction was discovered to be effective, and a pattern that was quite similar to this one was also identified in the antioxidant activities. Furthermore, enzyme inhibition and antioxidant activities were seen to a much larger degree in nonaqueous solvents in comparison to the water fraction and pure venom. This was the case. On the basis of the findings of the LC-MS/MS analysis, it was concluded that the water fraction mostly included the proteins. Although the small organic metabolite/peptide content of M. l. obtusa was not identified in the present study, considering the low or no cholinesterase and antioxidant activities of crude venom and water fractions (including mostly proteins), our results suggest that smaller minor organic compounds apart from proteins enriched in nonaqueous solvents (e.g., ethyl acetate, methanol) may contribute to the observed bioactivities. An example of this would be the presence of adenosine in snake venom, which also possesses antioxidant effects.^63,64^ It is possible that the knowledge of the mechanism of action of crude venoms may be expanded if proteins, peptides, and metabolites were identified simultaneously in the same venom and fractions.
